# ­­Eleven tips for operational researchers working with health programmes: our experience based on implementing differentiated tuberculosis care in south India

**DOI:** 10.1080/16549716.2022.2161231

**Published:** 2023-01-09

**Authors:** Hemant Deepak Shewade, Asha Frederick, Madhanraj Kalyanasundaram, Joshua Chadwick, G. Kiruthika, T. Daniel Rajasekar, K. Gayathri, R. Vijayaprabha, R. Sabarinathan, Shri Vijay Bala Yogendra Shivakumar, Kathiresan Jeyashree, P. K. Bhavani, S. Aarthi, K. V. Suma, Delphina Peter Pathinathan, Raghavan Parthasarathy, M. Bhavani Nivetha, Jerome G. Thampi, Deiveegan Chidambaram, Tarun Bhatnagar, S. Lokesh, Shanmugasundaram Devika, Timothy S. Laux, Stalin Viswanathan, R. Sridhar, K. Krishnamoorthy, M. Sakthivel, S. Karunakaran, S. Rajkumar, M. Ramachandran, K. D. Kanagaraj, M. Kaleeswari, V. P. Durai, R. Saravanan, A. Sugantha, S. Zufire Hassan Mohamed Khan, P. Sangeetha, R. Vasudevan, R. Nedunchezhian, M. Sankari, N. Jeevanandam, S. Ganapathy, V. Rajasekaran, T. Mathavi, A. R. Rajaprakash, Lakshmi Murali, U. Pugal, K. Sundaralingam, S. Savithri, S. Vellasamy, D. Dheenadayal, P. Ashok, K. Jayasree, R. Sudhakar, K. P. Rajan, N. Tharageshwari, D. Chokkalingam, S. M. Anandrajkumar, T. S. Selvavinayagam, C. Padmapriyadarsini, Ranjani Ramachandran, Manoj V. Murhekar

**Affiliations:** a ICMR – National Institute of Epidemiology, Chennai, India; b State TB Cell, Government of Tamil Nadu, Chennai, India; c ICMR – National Institute for Research in Tuberculosis, Chennai, India; d The WHO Country Office for India, New Delhi, India; e Tsehootsooi Medical Center, Fort Defiance, AZ, USA; f Department of Medicine, Jawaharlal Institute of Postgraduate Medical Education and Research (JIPMER), Puducherry, India; g Government Hospital of Thoracic Medicine, Tambaram, India; h Department of Respiratory Medicine, Tirunelveli Medical College Hospital, Tirunelveli, India; i Directorate of Medical and Rural Health Services, Government of Tamil Nadu, Chennai, India; j Directorate of Public Health and Preventive Medicine, Government of Tamil Nadu, Chennai, India

**Keywords:** Triaging for severe illness, ending TB deaths, differentiated TB care, operational research, technical support

## Abstract

Due to the workload and lack of a critical mass of trained operational researchers within their ranks, health systems and programmes may not be able to dedicate sufficient time to conducting operational research (OR). Hence, they may need the technical support of operational researchers from research/academic organisations. Additionally, there is a knowledge gap regarding implementing differentiated tuberculosis (TB) care in programme settings. In this ‘how we did it’ paper, we share our experience of implementing a differentiated TB care model along with an inbuilt OR component in Tamil Nadu, a southern state in India. This was a health system initiative through a collaboration of the State TB cell with the Indian Council of Medical Research institutes and the World Health Organisation country office in India. The learnings are in the form of eleven tips: four broad principles (OR on priority areas and make it a health system initiative, implement simple and holistic ideas, embed OR within routine programme settings, aim for long-term engagement), four related to strategic planning (big team of investigators, joint leadership, decentralised decision-making, working in advance) and three about implementation planning (conducting pilots, smart use of e-tools and operational research publications at frequent intervals). These may act as a guide for other Indian states, high TB burden countries that want to implement differentiated care, and for operational researchers in providing technical assistance for strengthening implementation and conducting OR in health systems and programmes (TB or other health programmes). Following these tips may increase the chances of i) an enriching engagement, ii) policy/practice change, and iii) sustainable implementation.

## Operational research: how it helps health systems and programmes

The health system, especially in low- and middle-income countries, is overwhelmed with a high patient load from dealing with multiple diseases within limited infrastructure and resources [1,2]. In these contexts, operational research (OR) defined as ‘any research producing practically usable knowledge (evidence, findings and information), which can improve programme implementation (e.g. effectiveness, efficiency, quality, access, scale-up, sustainability) regardless of the type of research (design, methodology and approach)’ [3], is essential to understanding what works and what does not in programme implementation [1,2]. The fundamental ideas embedded in the varied definitions of OR explain that it helps identify and resolve programme problems, provides evidence-based programme decisions to policymakers and programme managers and enhances the programme quality and effectiveness by applying scientifically validated methodologies [2–6]. OR somewhat mimics routine monitoring and evaluation. However, unlike regular monitoring and evaluation, it is hypothesis-driven; the hypothesis is tested using rigorous scientific procedures that enable analytical comparisons, allowing inferences to be utilised to improve policy and practice [3].

## Role of operational researchers from research/academic organizations

Embedding OR is the key to establishing an evidence-based national health programme [7–10]. Ideally, OR should be conducted by system/programme staff. Due to workload and lack of a critical mass of trained operational researchers within their ranks, health programmes and systems may not be able to dedicate sufficient time to conduct OR [4]. Until this happens through capacity-building initiatives [11], they may need the technical support of operational researchers from research/academic organisations [10]. Over the long term, operational researchers also start contributing towards strengthening and improving programme performance through implementing recommendations of OR. In some instances, the technical support units may be embedded within the health system through an arrangement; for example, World Health Organization (WHO) county office for India provides technical assistance to the national TB elimination programme (NTEP) through its technical support network [12,13].

## Tamil Nadu - *Kasanoi Erappila Thittam* (TN-KET)

Tamil Nadu (south India, population of ≈ 72 million) has 35 NTEP districts with the district population ranging from 0.6 to 4 million [14]. Tamil Nadu’s TB case notification rate (reported data, public and private combined) in 2021 was 100 per 100,000, with ≈4500 notifications per month from public facilities. The case fatality for patients diagnosed in 2020 was 6.4% compared to 3.4% for the 2019 cohort [15,16].

The central TB division’s guidelines recommend assessing all TB patients at diagnosis using 16 indicators (comprehensive assessment) [17]. However, comprehensive assessment requires diagnostic and clinical capacity in the facilities that diagnose TB, and not all facilities have similar capacity. Measurement of 16 indicators among all TB patients may require a referral and induce a possible delay [18]. In the neighbouring state of Karnataka, early deaths (within two months) were high (7%), and half of them occurred within two weeks [18]. Early TB deaths were 2.4 times more likely among patients with very severe undernutrition or respiratory insufficiency, or poor performance status at diagnosis (also called ‘high risk of severe illness) (Box 1) when compared to those without any one of these conditions [18–20]. Early deaths were as high as 14% among those with very severe undernutrition and 18% among those with poor performance status. Prior experience suggested this triage tool (Box 1) was feasible to use in programme setting (high triaging at diagnosis, a median one-day time interval between diagnosis and triaging and minimal missing data) [20,21].

**Table:** **Box 1.** Triaging tool for severe illness used in Tamil Nadu Kasanoi Erappila Thittam^ (TN-KET), India [19,20]*.

**If at least one of the following is present**, **then the person with TB is ‘high risk of severe illness’ (requires referral for comprehensive assessment and inpatient care)** Body mass index (BMI) less than or equal to (≤) 14.0 kg/m^2#^ BMI less than or equal to (≤) 16.0 kg/m^2^ with leg swelling^#^ Respiratory rate more than (>) 24 per minute^##^ Oxygen saturation less than (<) 94%^##^ Not able to stand without support (standing with support/squatting/sitting/bed ridden)

TB-tuberculosis; ^means TB death-free project in Tamil language; *Reprinted from Shewade HD et al [20] under a CC BY licence, with permission from MDPI, Copyright MDPI 2021, tool adapted from Bhargava A et al [19]; ^#^very severe undernutrition indicators; ^##^respiratory insufficiency indicators.

Hence, we decided to introduce this triage tool at diagnosis before comprehensive assessment. We prioritised adults (≥15 years) with TB (not known to be drug resistant) from all public facilities with very severe undernutrition or respiratory insufficiency or poor performance status at diagnosis for comprehensive assessment and inpatient care. We included them irrespective of the TB site (pulmonary and extrapulmonary), type of confirmation (microbiologically and clinically confirmed) and previous history of treatment (newly and previously treated patients). Microbiological confirmation was done through smear microscopy, Xpert MTB/Rif®, Truenat MTB®, Truenat MTB-RIF® and line probe assay, among others.

**Figure 1.**
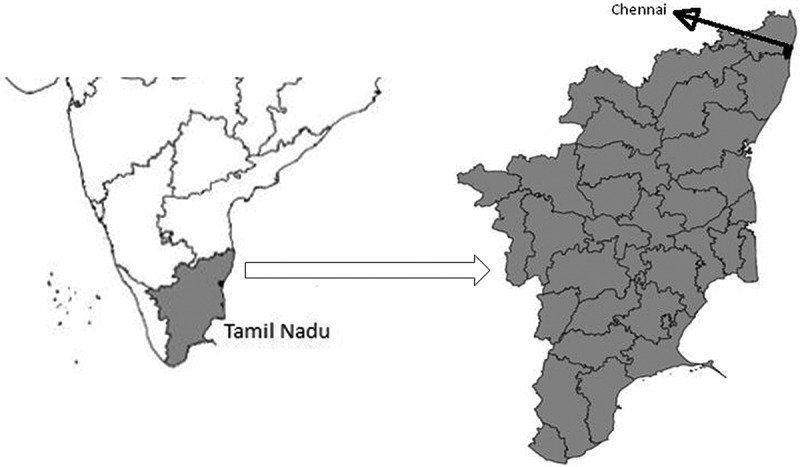
Map of India showing Tamil Nadu and its 30 NTEP districts that were included in TN-KET (2022)*.

TN-KET: Tamil Nadu *Kasanoi Erappila Thittam* - means TB death-free project in the Tamil language; NTEP: National TB elimination programme; there are 35 NTEP districts in Tamil Nadu, of which 30 were included in TN-KET (five districts from Chennai, the capital city, were not included).

We are sharing our experience in implementing this differentiated TB care model to reduce TB deaths in the state. Starting April 2022, we implemented Tamil Nadu - *Kasanoi Erappila Thittam* (TN-KET), meaning ‘TB death free project’ in the Tamil language, in all NTEP districts (n = 30) in the state, except five NTEP districts in Chennai – the capital city (Figure 1). The project included a preparation phase (December 2021 to March 2022), a two-week pilot (14–27 March 2022) and the implementation phase (April 2022 onwards). There is an understanding that it will be implemented for at least two years.

TN-KET is an implementation project with an OR component incorporated from the start. We implemented TN-KET as a routine activity under Tamil Nadu’s NTEP. The OR focussed on assessing/understanding i) how many severe patients were missed by the triage tool, ii) feasibility (losses and delay in TN-KET care cascade and factors associated) and impact of TN-KET (reduction in TB deaths) and iii) patient and provider perspectives into enablers, challenges and suggested solutions to improve TN-KET performance.

In TN-KET, we introduced a simple and easy-to-use paper-based triage tool in all the public facilities diagnosing TB in the 30 districts of the state – primary health centre and above (Box 1) [19,20]. We prioritised ‘high risk of severe illness’ patients for comprehensive assessment at one of the state’s 150 nodal inpatient care facilities, each with a nodal physician (internal medicine or chest specialist). These were identified by the state TB cell in consultation with the Directorate of Medical and Rural Health Services and the Directorate of Medical Education, Tamil Nadu. Referral from diagnosing public facility to nodal inpatient facilities was done using public ambulance services. We introduced a paper-based case record form in the 150 nodal inpatient care facilities. These records shall streamline the comprehensive assessment, calculation of the total score after the comprehensive assessment, confirmation of severe illness (see Box 2 for the criteria) and identification of the issues that need attention during inpatient care. We earmarked 900 beds for TB and developed an ‘inpatient care guide for adults with TB who are severely ill’ (for use by the nodal physician) [22].

**Table:** **Box 2.** Criteria for patients requiring inpatient care* among adults (≥15 years) with TB based on CTD 2021 technical guidance on differentiated care of TB patients in India [17].

**Criteria**		Considered to be an emergency**	**Scoring criteria**	**Score assigned**
1	Pulse rate (per minute)		<60 >100 (persistent after 30 minutes)	2 2
2	BMI (kg/m^2^)	Yes (<14)	<14 <16 with pedal oedema >40	1 1 1
3	MUAC (cm)		<16	1
4	Temperature (Celsius)	Yes (<35, >41)	<35 >41	2 2
5	Blood pressure (mm Hg)	Yes	Hypertension (≥140/90) Hypotension (diastolic below 60)	2 2
6	Respiratory rate (per minute)	Yes (>24)	18-24 25-30 >30	1 2 3
7	Oxygen saturation (%)	Yes (<94)	90-93 85-90 <85	1 2 3
8	Haemoglobin (g%)	Yes (<7)	<7	2
9	Icterus		Present	1
10	Pedal Oedema		Present	1
11	General condition	Yes (if unable to walk, drowsy, unconscious)	Inability to walk but conscious and oriented Conscious, not oriented Drowsy	1 2 3
12	HIV		Positive and on ART Positive and not on ART	1 2
13	Random blood sugar		<70 >200	2 2
14	Total white blood cell count		TC > 11000 TC < 4000	1 1
15	Chest radiograph	Yes (massive pneumothorax, hydropneumothorax)	Hydropneumothorax Bilateral consolidation	3 2
16	Haemoptysis	Yes	Present	3

TB – tuberculosis, CTD – central TB division, BMI – body mass index, MUAC – mid upper arm circumference, HIV – human immunodeficiency virus, ART – antiretroviral treatment.

*Patients with very severe undernutrition, respiratory insufficiency or poor performance status are prioritized for comprehensive assessment. A total score of more than one – to be provided inpatient care, a total score of more than three – to be provided inpatient care in a facility with an intensive care unit. **If an indicator suggests an emergency, inpatient care should be provided, irrespective of the total score.

*Modifications to the criteria provided by CTD 2021 technical guidance: blood sugar of > 128 replaced with >200. In addition, inpatient care may be provided if the treating physician feels the need for inpatient care.

**Figure 2.**
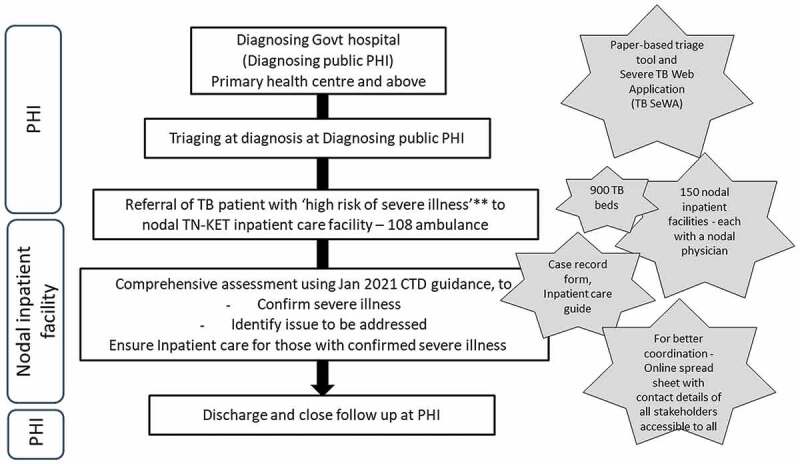
Tamil Nadu Kasanoi Erappila Thittam* (TN-KET) care cascade, Tamil Nadu India.

**Figure 3.**
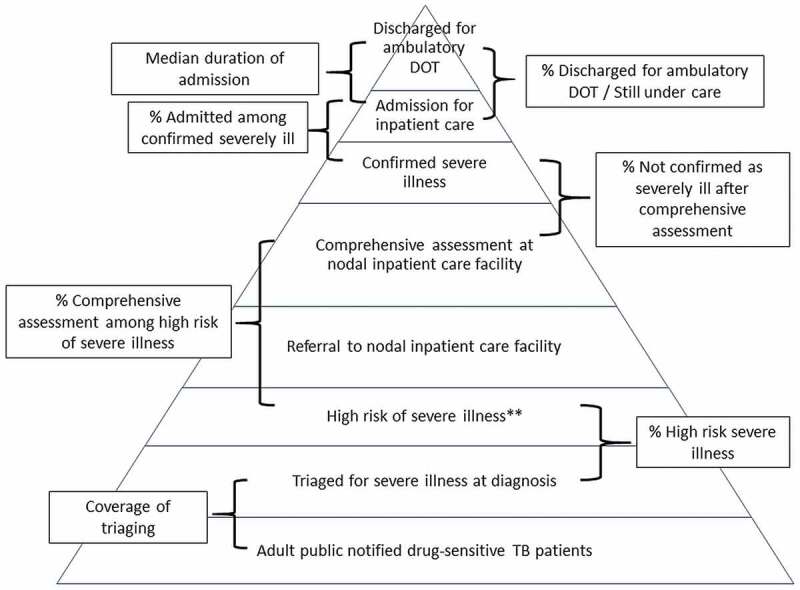
Indicators used in Tamil Nadu Kasanoi Erappila Thittam* (TN-KET), India.

We used a Severe TB Web Application (TB SeWA, s*ewa* means service in Sanskrit) to track the TN-KET care cascade (Figure 2) [23]. The monitoring indicators used in TN-KET are depicted in Figure 3.

PHI – peripheral health institution, TB – tuberculosis, CTD – Central TB division, *means TB death-free project in the Tamil language, **patients with very severe undernutrition or respiratory insufficiency or poor performance status.

*means tuberculosis death-free project in Tamil language, **patients with very severe undernutrition or respiratory insufficiency or poor performance.

## Our eleven tips

We have divided these 11 tips into three categories: broad principles (n = 4), strategic planning (n = 4) and implementation planning (n = 3) (Box 3). These may act as a ‘how we did it’ guide for other Indian states and countries with high TB burden that are targeting to end TB deaths. Globally and nationally, beyond the pilots assessing the feasibility of triaging for severe illness at diagnosis, there is limited experience in implementing the differentiated TB care cascade holistically in routine TB programme settings [18,20,21]. For operational researchers interested in providing technical assistance for strengthening implementation and conducting OR in health systems and programmes (TB or other health programmes), following these tips will increase the chances of i) an enriching engagement, ii) policy/practice change based on findings of OR and iii) sustained implementation after the organisation providing technical support phases out. Kumar AMV et al. and Harries AD et al. have discussed in detail how national TB programmes may integrate and build a culture of OR and use OR to end TB [5,24]. Here, we have shared our learnings in the form of eleven tips and relevant examples under each of what we did in TN-KET (see Box 3).

**Table:** **Box 3.** Eleven tips for operational researchers working with health programmes: our experience based on implementing Tamil Nadu Kasanoi Erappila Thittam* (TN-KET), India.

**Tips for an operational researcher**		**What we did in TN-KET**
***Broad principles***		
1	Design the OR on priority areas and make it a health system initiative	TN-KET focuses on reducing TB deaths and is implemented as a health system initiative involving the key directorates under the state health department.
2	Implement simple and holistic ideas	We made significant efforts to provide enabling environment to complete the care cascade (Figure 2) by identifying nodal inpatient care facilities, and nodal physicians and sensitizing them with an ‘inpatient care guide for adults with TB who are severely ill’. TN-KET is simple in a sense the data capture (paper-based and in e-tool) is limited to the variables essential for programmatic monitoring
3	Embed the new intervention and the OR within routine programme settings	The state and districts included the TN-KET monitoring indicators (Figure 3) in the routine monthly/quarterly reports, state internal evaluations and review meetings of the broader programme (NTEP in this case).
4	Aim for long-term engagement and not ‘one off.’	We intend to work together for two years
***Strategic planning***		
5	A big team of investigators	All the partners, including all district-level programme managers, have been involved as a co-investigator (n > 60) under TN-KET’s OR component
6	Role of joint leadership	The state TB officer of Tamil Nadu and a senior Scientist (expert in operational research) from ICMR-NIE jointly lead TN-KET
7	Decentralized decision-making	Decentralizing decision-making at district-level through the technical support unit. At ICMR-NIE-level, weekly internal review meetings for technical supporters. In addition to updating the status of the districts under them, new ideas are brainstormed from time to time. Supportive supervision visits by technical supporters
8	Work well in advance	All the activities in the preparation phase (December 2021 to March 2022) of TN-KET were started well in advance including capacity building and training for state-level and district-level NTEP managers We informed all the 30 districts well in advance about deadline for the start of pilot and implementation phases
***Implementation planning***		
9	Conduct pilot study	Large-scale pilots instead of small scale Report submitted and discussed at a state-level review meeting
10	Smart use of e-tools for sharing material, data capture and monitoring	The district TB cells are provided with three sets of e-tools. 1. TN-KET tools folder -shared over Google Drive 2. Severe TB Web Application (TB SeWA)- a simple case-wise web-based application to track the TN-KET care cascade (Figure 2) 3. an online monitoring tool – a spreadsheet stored in Google Drive
11	Plan operational research publications at frequent intervals	We identified various manuscripts across the study period, the lead and the deadline for submission

NTEP: National TB elimination programme; OR: operational research; ICMR-NIE Indian Council of Medical Research – National Institute of Epidemiology; *means TB death free project in Tamil language.

## Broad principles

### Tip 1: design the OR on priority areas and make it a health system initiative

Reducing TB deaths by 90% in 2030 and 95% in 2035 (compared to 2015) are the global targets under the WHO’s End TB strategy [25]. India has set an ambitious deadline of a 90% reduction by 2025, five years before 2030 [13]. For the first time in a decade, there was an increase in estimated TB deaths by 7% in 2020 due to COVID-19 (compared to 2015) [26]. COVID-19 and NTEP’s 2021 guidance on differentiated TB care was a window of opportunity to implement TN-KET [17].

To ensure that the new health intervention addressing a priority area succeeds, wherever possible, it should be envisaged as a health systems initiative rather than a project of the specific health programme. The Tamil Nadu state TB cell (state-level programme management) and the Indian Council of Medical Research-National Institute of Epidemiology (ICMR-NIE – Government research organisation) led TN-KET with support from the WHO Country Office for India and the ICMR-National Institute for Research in Tuberculosis (ICMR-NIRT). This model was facilitated as a health system initiative by the National Health Mission Tamil Nadu; Directorate of Medical and Rural Health Services, Tamil Nadu; Directorate of Medical Education, Tamil Nadu; and Directorate of Public Health and Preventive Medicine, Tamil Nadu (policymakers).

### Tip 2: implement simple and holistic ideas

Many health system interventions/solutions fail because of a lack of foresight in preparing and planning for the entire care cascade. The question of ‘what next?’ is often not thought out in detail. In addition, the mantra should be to keep the larger picture in mind. The focus should be on simple and doable ideas that can be systematically implemented for all patients (ensuring high coverage), keeping in mind the available resources in the health system. TN-KET was holistic because we ensured follow-up during the entire care cascade. TN-KET is simple because the data captured (paper-based and in e-tool) was limited to the variables essential for district-level and state-level programmatic monitoring (see tip 10 for details).

### Tip 3: embed the intervention and the OR within routine programme settings

Embedding OR into health programme settings may act as an intellectual stimulus for the programme implementers/managers [4]. Suppose the OR is around a new intervention at the state-level. In that case, the key is to ensure that the novel intervention (TN-KET) is implemented concurrently with the health programme in routine programme settings, with monitoring and evaluation indicators interwoven with the broader health programme (the state NTEP). It is practical to utilise the existing supportive supervision framework of the state NTEP, which could be aided by the academic/research organisation’s support. The state and districts included the TN-KET monitoring indicators (Figure 3) and checklist in the routine monthly/quarterly reports, state/programme internal evaluation visits and review meetings of the state’s NTEP.

### Tip 4: aim for long-term engagement and not ‘one-off’

Engagement between operational researchers and health systems/programmes may be one-off or long term. Programme managers appreciate if the operational researchers show genuine interest, build rapport and engage over the long term, thus providing maximum opportunity for policy/practice change leading to implementation strengthening and improved programme outcomes. Long-term engagement offers a better understanding of the political dimensions of health policy. It also helps in conducting realistic research, evaluation, and anticipation of opportunities and constraints [27]. In TN-KET, we envisaged and conceptualised the project with the intent to work together for two years, at least.

## Strategic planning

### Tip 5: a big team of investigators

Good OR is collaborative research and most useful where programme implementers play a part in problem identification, planning, design and conduct of OR (‘involve them from the start’). The key is to collaborate with all the potential stakeholders and not hesitate to offer the role of co-investigator to state and district-level implementers. This practice fosters collaboration and instils ownership and motivation [28]. We recommend that the team comprise policymakers, programme administrators, key implementers and operational researchers from research/academic organisations.

Under TN-KET’s OR component, we involved all partners including all district-level programme managers, as co-investigator. We formed a core-working group for crucial decision-making and performing key activities. This core group involved the principal investigator(s) and the technical support unit from ICMR-NIE that provided technical support to districts. They worked closely with WHO TB consultants and provided updates and recommendations from time to time. The trio of programme managers, operational researchers and WHO TB consultants worked well.

### Tip 6: role of joint leadership

It would be ideal for programme managers to play a leadership role in OR [29]. Joint leadership with operational researchers from research/academic organisations often motivates and empowers programme managers. This way, programme managers and their teams generate feedback from the frontlines while researchers provide the rigour and expertise needed for robust studies. The proactive role of operational researchers is often necessary for the initial stages of implementation. A critical point in TN-KET was that the state-level NTEP manager (state TB officer) of Tamil Nadu and a senior medical scientist (expert in operational research) from ICMR-NIE jointly led the project. This way there was ownership and accountability from both sides (implementers/programme managers and operational researchers from research organisations).

### Tip 7: decentralised decision-making

Decentralisation is promoted as a strategy to improve health system performance by bringing decision-making closer to service delivery [30–32]. If everyone works following the broader framework with sufficient decentralisation, we can generate and implement new ideas. In TN-KET, we decentralised decision-making in the technical support unit (medical scientists, technical officers and consultants from ICMR-NIE, ICMR-NIRT and WHO country office) and the districts linked to a person(s) in the technical support unit. At the ICMR-NIE level, we conducted weekly internal review meetings for the technical support unit, where we received updates on districts’ status and brainstormed new ideas. Keeping the long-term picture in mind, the technical supporters built the capacity of the district-level NTEP managers and made supportive supervision visits. By December 2022, we had covered all districts at least once.

### Tip 8: work well in advance

Working well in advance is good practice for the success of any activity. As we began early in December 2021 with the activities in the preparation phase (see S1 Annex), this strategy provided adequate time for all the stakeholders to provide feedback. Initially, for example, we had planned to implement only the triaging component in the first three months (April to June 2022), and starting July 2022, we were considering working with the health system for referral, comprehensive assessment and inpatient care. During the brainstorming in the preparation phase, we received the suggestion from Tamil Nadu National Health Mission to implement all steps in the TN-KET care cascade starting in April 2022. They assured necessary administrative support for this.

From the beginning, we informed the 30 districts about the two critical milestones related to the start of TN-KET: initiation of the pilot (14 March 2022) and its implementation (1 April 2022). The mantra is ‘start early – do not hurry’.

## Implementation planning

### Tip 9: conduct a pilot study

Before implementing any large-scale intervention, it is essential to pilot. The issue with small-scale pilots is that requirements of large-scale implementation are rarely considered [33]. In TN-KET, we began keeping the end in mind [33]; instead of piloting in a few districts, we piloted all the steps of the TN-KET care cascade (Figure 2) for two weeks in all 30 NTEP districts. District-level NTEP managers led all the activities. We wanted the districts and state NTEP teams and technical support unit to get a ‘feel’ of TN-KET. We presented and discussed the report of the pilot at a state-level review meeting in April 2022.

### Tip 10: smart use of e-tools for sharing material, data capture and monitoring

Establishing and maintaining a sound data-sharing and management system are essential. The e-tools must be without any ambiguity and easy for everyone to use. The mere availability of e-tools should not be a reason to collect everything ‘under the sun’ and overburden the staff. The mantra is to ‘keep it simple’. Another issue plaguing the health system is skewed data collection/reporting efforts and neglecting patient care. Hence, it must be conveyed to the care providers that patient care is their primary responsibility, not merely filling e-tools. Delays in filling e-tools should be tracked and accepted if patient care is not affected. Keeping these points in mind, we developed and provided three sets of e-tools to the district TB cells.

First, we shared the TN-KET tools folder containing project-related files (e.g. paper-based triage tool, paper-based case record form, and inpatient care guide) over Google Drive (see S2 Annex). We requested districts to download the corresponding file from the TN-KET tools folder and discouraged them from storing copies in the computers. This practice ensured that the updated versions were constantly in use.

Second, we used a simple case-wise tracking e-tool (TB SeWA) to track the patients in the TN-KET care cascade. We sensitised during training that the paper-based triaging-related data collection (five additional indicators-Box 1) at diagnosis in a public facility should be clubbed along with the routine baseline data collection of TB patients. Data entry access in TB SeWA was restricted to sub-district-level staff (that notify and supervise TB treatment), and this was clubbed along with their entering baseline patient details in NIKSHAY (an electronic case-based TB notification portal) [34]. Post-triaging data (post-referral and post-admission) in TB SeWA included simple ‘yes/no’ answers. We captured the dates only for triaging, referral, admission and discharge. We included only the variables required for monitoring TN-KET. In addition, we repeatedly emphasised during training that action (completion of care cascade) was far more critical than capturing post-triaging data in TB-SEWA. The web application had a dashboard that showed the aggregate numbers related to critical points in the care cascade required for generating TN-KET monitoring indicators. TB SeWA also had the option to view and download all case-wise district data for the district programme managers. The TB SeWA user ID provided at the sub-district and district levels was similar to the one used in NIKSHAY. We also developed a systematic mechanism for troubleshooting or resolving queries related to TB SeWA.

In TB SeWA, we did not duplicate data capture of baseline characteristics (routinely captured in NIKSHAY at the sub-district level) and other follow-up data (programme-reported deaths and treatment outcomes routinely captured in NIKSHAY). The date of notification was captured in TB SeWA as it was required to generate the monthly monitoring indicators. For in-depth analysis in the OR papers, we merged case-wise data of TB-SeWA with NIKSHAY using the NIKSHAY unique identifier. Hence, we smartly utilised routinely captured data of NIKSHAY for the OR.

Third, we developed an online monitoring tool (a spreadsheet stored in Google Drive) with four sheets. Sheet one consisted of district-wise contact details of all the sub-district-level staff, district-level TB staff, nodal physicians and district technical supporters from ICMR-NIE, ICMR-NIRT and WHO India. This tool contributed towards better coordination of to and fro referrals (from diagnosing facility to nodal inpatient care facility to back to the patient’s residence for ambulatory DOT), especially out-of-district referrals. We adopted this practice in line with lessons learnt from China [35]. Among TB patients transferred out using China’s web-based TB information management system, the chances of non-evaluation of treatment outcome increased if the transfer out was to a geographically distant location compared to within the county (TB administrative unit). Based on this information, China’s TB programme recommended regularly updating the county-level nodal person’s contact details within the web-based TB information management system [35].

Sheet two was used to monitor activities across the project phases – preparation, pilot and implementation. Sheet two had questions (with self-reported ‘yes/no’ answers by the district programme manager) to assess the progress of activities (see S3 Annex). The idea was to enable the district programme managers to compare their status with other districts, motivating them to improve their performance.

In sheet three, the districts filled monthly aggregate numbers of total TB deaths, early TB deaths and home deaths. Once this was entered, the figures containing the trend lines for monthly/quarterly proportion of home/early deaths among all TB deaths (surrogate indicators) were auto-generated. If districts/states met the ’80-80-80-7’ target (80% triaging, 80% ‘high risk of severe illness’ referred, comprehensively assessed and confirmed as severely ill, 80% confirmed patients admitted and median seven days of inpatient care) and the trend lines did not show a downward trend, the districts/state reviewed the quality of inpatient care. Sheet four had TN-KET’s timeline (Gantt chart).

### Tip 11: plan operational research publications at frequent intervals

It is wise to identify the broad areas and titles of publications well in advance and divide those into short-, medium- and long-term outcomes. The leadership roles for each of these may be identified and intimated well in advance. Outcomes in the form of publications at frequent intervals keep everyone motivated. While we do this, we must ensure that all the authors fulfil the international committee of medical journal editors’ authorship criteria [36]. In TN-KET, we did this through an internal meeting of the core-working group at ICMR-NIE and shared this idea with the state TB office.

There is no doubt that OR must lead to research publications, and the number of publications is one of the objective outcomes of OR. Beyond the publications, translating generated knowledge into policy/practice is essential [37]. As discussed before, in TN-KET, the OR papers were the output of in-depth individual-level data analysis after merging the TB SeWA and NIKSHAY databases. These provided additional information that is beyond the scope of routine monitoring and evaluation. This included i) delays in triaging and admission of severely ill patients, ii) predictors of losses in TN-KET care cascade, iii) systematic qualitative enquiry into the ‘why’ and ‘how’ of quantitative findings, and iv) rigorous analysis of the impact of TN-KET on mortality. As there was a close working relationship between operational researchers and the state, this information was shared as reports with the state even before publication. Feedback was sought, and then recommendations were discussed and implemented. Hence, from time to time, the OR papers guided the strategies being implemented under TN-KET.

## Conclusion

This article describes our experience implementing India’s first ever-statewide differentiated TB care model with an inbuilt OR component. Through this ‘how we did it’ paper, we have provided eleven tips for operational researchers, health programme managers/implementers and policymakers to successfully conduct OR and contribute towards strengthening the implementation of existing interventions in place. Following these tips may increase the chances of a fruitful engagement of operational researchers with health systems/programmes for policy/practice change. The relevant examples of what we did in TN-KET under each tip may be a guide for other states and high TB-burden countries devising similar interventions to end TB deaths. However, there are potential limitations to operationalising these tips for differentiated TB care in some low- and middle-income countries: i) non-availability of internal medicine or chest specialist in secondary and tertiary-level facilities, ii) relatively long distance between diagnosing facilities or patient residences and these secondary and tertiary-level facilities iii) non-availability of an assured public ambulance service. Context-specific modifications may still be required.

## Supplementary Material

Supplemental Material

## Paper context

We implemented a statewide-differentiated TB care strategy and the operational research around it in Tamil Nadu, south India, targeted towards reducing TB deaths. The state TB programme and a national public research organisation jointly led this. In this capacity-building paper, based on our experience, we have shared eleven tips for operational researchers. Following these tips may increase the chances of a fruitful engagement with health systems/programmes. Against each tip, we have shared example(s) from our experience.
