# Supplementary material for: ­­Eleven tips for operational researchers working with health programmes: our experience based on implementing differentiated tuberculosis care in south India
Source: Glob Health Action. 2023 Jan 9;16(1):2161231. doi: 10.1080/16549716.2022.2161231 (PMC9833404; doi:10.1080/16549716.2022.2161231)
Supplement: Supplemental Material [file ZGHA_A_2161231_SM7343.docx]

**S1 Annex. Steps in preparation phase of Tamil Nadu *Kasanoi Erappila Thittam** (TN-KET), December 2021 to March 2022**

| 1 | Development of proposal, triaging tool, inpatient care guide, web application for data capture, e-tools for monitoring, training material, standard operating procedures and short videos (<2 min) related to collecting triaging data for circulation with all diagnosing facilities |
| --- | --- |
| 2 | Identification of nodal physicians and nodal TN-KET facilities for inpatient care |
| 3 | Conducting training   - Training planned and carried out at multiple levels. - The state-level training of trainers (state-level NTEP managers from state TB cell and district TB cells of all 30 districts, technical supporters from ICMR Institutes and WHO) was conducted in December 2021 in hybrid mode. - During January-February 2022, thirty districts were divided into six clusters (five districts in each cluster), and cluster-level online training was given to district-level programme managers and implementers (up to field-level staff). - Again, a refresher training of all district-level implementers was conducted in person at the district-level, led by district NTEP programme manager (district TB officer). |
| 4 | Monitoring the preparation of districts to implement TN-KET (see **S2 Annex**) |
| 5 | Obtaining scientific, ethical and administrative approvals |

*means tuberculosis death free project in Tamil language; NTEP: National TB elimination programme; TB: tuberculosis; ICMR: Indian Council of Medical Research; WHO: World Health Organization

**S2 Annex. Contents of the TN-KET tools folder (shared over Google Drive)**

| 1 | **Paper-based triaging tool** |
| --- | --- |
| 2 | **Case record form for comprehensive assessment of ‘high risk of severe illness’ patient at TN-KET nodal inpatient facility** |
| 3 | **Inpatient care guide for severely ill adults with TB – for use by nodal physician at TN-KET nodal inpatient facility** |
| 4 | **PowerPoint presentations (one short and one long) for orientation and refresher training** |
| 5 | **Standard operating procedures (short videos and documents) for orientation and refresher training** |
| 6 | **TN-KET indicator calculator (Excel sheet) and slide (PowerPoint)** |
| 7 | **Table containing distribution of districts among nodal persons from the technical support unit (scientists, technical officers and consultants from ICMR-NIE, ICMR-NIRT and WHO country office).** |
| 8 | Monthly and quarterly reports containing state and district wise TN-KET monitoring indicators (see **Figure 3**) |

TN-KET: Tamil Nadu *Kasanoi Erappila Thittam,* means TB death free project in Tamil language; ICMR: Indian Council of Medical Research; NIE: National Institute of Epidemiology; NIRT: National Institute for Research in Tuberculosis; WHO: World Health Organization

**S3 Annex. Questions (with self-reported ‘yes/no’ answers by district programme manager) in the online monitoring tool to assess the statewide, district wise progress of activities across the TN-KET phases, Tamil Nadu, India**

| 1. **Preparatory phase (December 2021 to March 2022)** | |
| --- | --- |
| 1 | Has the DPC been trained? |
| 2 | The proposed date for district-level training (to all district NTEP staff including all STS) to be conducted by DPC/DTO (if training is completed, please mention the date of training) |
| 3 | Has DPC informed district NTEP staff (including all STS) about project related training in the district (this is the training conducted within the district by DTO/DPC)? |
| 4 | Have ALL the STS been trained in the district (in district-level training conducted by DPC/DTO) |
| 5 | DPC / DTO in the district have web link of the TB SeWA along with the username and password |
| 6 | DPC / DTO in the district are able to log into TB SeWA? |
| 7 | ALL the STS in the district have link of the TB SeWA along with the username and password |
| 8 | ALL the STS in the district are able to log into TB SeWA? |
| 9 | DPC/DTO and STS have set a unique password for their TB SeWA account? *At first login, all users have to change the password* |
| 10 | Have ALL the STS in the district received paper-based data collection tool (English) and SOP document (English and Tamil) - soft copy |
| 11 | Have ALL the STS in the district shared copies of paper-based data collection tool (English) and SOP (English and Tamil) - soft copy, to ALL public PHIs |
| 12 | Whether all public PHIs have sufficient print out of paper-based data collection tool ('two page-single paper', front and back print) |
| 13 | Have SOP videos been shared with ALL STS in the district |
| 14 | Have SOP videos been shared with ALL public PHIs in the district |
| 15 | Have all public PHIs been sensitized /trained regarding triaging for severe illness, filling paper-based tool and taking local action? |
| 16 | Have the nodal person(s) of ALL public PHIs installed the N-TB App (used to calculate BMI)? |
| 17 | Local mechanisms to ensure high coverage of triaging in high burden facilities has been identified? |
| 18 | Are all the STSs clear about the additional variables (referral related) that need to be collected for people with 'high risk of severe illness'? Whether they can view them in TB SeWA? |
| 19 | District NTEP staff have access to the list of nodal inpatient care facilities in their district and contact details of nodal medical and paramedical staff of these facilities (provided in online monitoring tool)? This has been discussed and mechanisms of referral identified.0 |
| 20 | Is the district ready for pilot data collection? Response to all the previous questions must be 'yes'. [On or before 11 Mar 2022] |
| **b.** **Pilot (14-27 March 2022) and Implementation phase (starting from 1^st^ April 2022), once every week:** | |
| 1 | In his/her district, has DPC prepared the TN KET monitoring slide? Has appropriate action been taken after cross-checking? For example, week starting 18 Apr 2022, tracking patients diagnosed/notified during 1-17 April |

TN-KET: Tamil Nadu *Kasanoi Erappila Thittam* - means TB death free project in Tamil language; DTO: district TB officer (district-level NTEP manager); DPC: district programme coordinator; STS: senior treatment supervisor; PHI: peripheral health institution; SOP: standard operating procedure; TB SeWA: severe TB web application; * District programme managers are expected to respond once every week that they have calculated the indicators (cumulatively for that month) and have taken appropriate action
